# Individual variability in preference for energy-dense foods fails to predict child BMI percentile

**DOI:** 10.1016/j.physbeh.2017.03.047

**Published:** 2017-07-01

**Authors:** Christina Potter, Rebecca L. Griggs, Danielle Ferriday, Peter J. Rogers, Jeffrey M. Brunstrom

**Affiliations:** Nutrition and Behaviour Unit, School of Experimental Psychology, University of Bristol, 12a Priory Road, Bristol, BS8 1TU, UK

**Keywords:** Eating behavior, Energy density, Child, Parent, Food choice

## Abstract

Many studies show that higher dietary energy density is associated with greater body weight. Here we explored two propositions: i) that child BMI percentile is associated with individual differences in children's relative preference for energy-dense foods, ii) that child BMI percentile is associated with the same individual differences between their parents. Child-parent dyads were recruited from a local interactive science center in Bristol (UK). Using computerized tasks, participants ranked their preference and rated their liking for a range of snack foods that varied in energy density. Children (aged 3–14 years, *N* = 110) and parents completed the tasks for themselves. Parents also completed two further tasks in which they ranked the foods in the order that they would prioritize for their child, and again, in the order that they thought their child would choose. Children preferred (*t*(109) = 3.91, *p* < 0.001) and better liked the taste of (*t*(109) = 3.28, *p* = 0.001) higher energy-dense foods, and parents correctly estimated this outcome (*t*(109) = 7.18, *p* < 0.001). Conversely, lower energy-dense foods were preferred (*t*(109) = − 4.63, *p* < 0.001), better liked (*t*(109) = − 2.75, *p* = 0.007) and served (*t*(109) = − 15.06, *p* < 0.001) by parents. However, we found no evidence that child BMI percentile was associated with child or parent preference for, or liking of, energy-dense foods. Therefore, we suggest that the observed relationship between dietary energy density and body weight is not explained by individual differences in preference for energy density.

## Highlights

• Higher dietary energy density (ED) is associated with greater body weight.
• A novel, computerized task was implemented to assess preference for ED.
• Children's and parent's relative preferences for ED were measured.
• Variation in relative ED preference was not associated with child BMI percentile.
• Alternate explanations of the association between dietary ED and BMI are discussed.

## 1 Introduction

The modern Western diet is often characterized by the widespread availability of highly palatable and energy-dense foods. Calorie for calorie, energy-dense foods are expected to deliver relatively less satiety [1] and may be selected in larger portions (in total calories) for this reason [2]. Larger portions also promote an increase in meal size [3] and the combined effect of portion size and energy density can have a dramatic impact on energy intake, both in adults [4], [5] and in children [6]. In addition, energy-dense foods tend to be less expensive, which promotes their selection [7].

Many studies show that the consumption of energy rich foods is associated with higher body weight (for a review see [8]). This relationship has been demonstrated across ethnic groups [9] and in both children [10] and adults [11]. In children this is a particular concern because eating habits are established early in life [12], [13] and childhood obesity greatly increases the risk of being overweight in adulthood [14]. Evidence from longitudinal studies shows that dietary energy density is a risk factor for greater adiposity in childhood [15] and that this relationship is preserved into adolescence [16].

The relationship between dietary energy density and adiposity might reflect variation in the availability of these foods [17] or perhaps personal beliefs about the need to restrict their consumption [18]. In this study, we consider an additional proposition that childhood adiposity is associated with individual differences in relative preference for energy dense foods. Children are born with an innate liking for sweetness [19]. However, they also learn to prefer energy-dense foods based on an association that forms between the post-ingestive effects of a food and its sensory characteristics [20], [21], [22], [23]. Social learning and peer modelling also play a critical role in further modifying preferences [24]. By the age of five, children already show a very clear relative preference for fruits and vegetables that are high in energy density [25]. One possibility is that some children express a refined ability to discriminate between foods based on their energy density, and select foods on this basis. However, before transitioning into adulthood, a child's diet is often determined by caregivers [26]. Parents were children once and may themselves differ in the extent to which their choices are governed by a learned preference for energy-dense foods. Therefore, a second proposition is that childhood adiposity is associated with this individual parental difference.

To evaluate these hypotheses we developed a novel food-choice task that involved ranking various snack foods by preference. The task was completed separately by children and their parents in response to several lunchtime scenarios. To complement these measures, children and parents also rated their liking for each food. We were interested to assess individual differences in preference for and liking of energy-dense foods. The aim of the study was to explore whether these differences are associated with child BMI percentile. In turn, this might help to explain the observed relationship between dietary energy density and child BMI percentile.

## 2 Method

### 2.1 Participants

An opportunity sample of child-parent dyads (*N* = 130) were recruited from a local interactive science center in Bristol (UK). All children were English-speaking and aged between 3 and 14 years. Children with food allergies or intolerances were excluded, together with vegetarians and vegans. The study protocol was approved through the local Faculty of Science Human Research Ethics Committee. Financial compensation was not provided.

### 2.2 Photographic stimuli

Our photographic stimuli comprised nine snack foods of varying energy density. After consulting a dietician, we selected foods that are likely to be well-known and well-liked by children and parents in the UK. The snack foods were: 1) apple, 2) dried apricots, 3) banana, 4) cheese, 5) chocolate chip cookies, 6) grapes, 7) chocolate wafer bar, 8) potato chips, and 9) yogurt. Each food was photographed in its standard serving size and with packaging (if applicable). All pictures were taken using a high-resolution digital camera. Particular care was taken to ensure identical lighting in each photograph. The name of the food was included in the top left-hand corner of each image. See Table 1 for the macronutrient composition of each food.

**Table 1:** Macronutrient composition and liking of snack foods. For all liking ratings, *N* = 110.

Snack food	Macronutrient composition/100 g					Energy density (kcal/g)	Displayed weight (g)	Displayed energy (kcal)	Child liking (mean ± SD)	Parent liking (mean ± SD)
	Protein (g)	Carbohydrate (g)	Fat (g)	Fiber (g)	Salt (g)					
Apple	0.4	11.8	0.1	1.8	–	0.47	154	72.4	69.3 ± 26.8	73.5 ± 22.1
Apricots, dried	3.9	36	0.6	6.3	–	1.78	75	133.5	52.1 ± 35.9	58.7 ± 31.4
Banana	1.2	23	< 0.5	1.1	< 0.5	1.03	118	121.5	60.1 ± 33.7	75.3 ± 22.8
Cheese (Cheestring©)	23	2.5	22.5	–	1.9	3.04	20	60.8	53.6 ± 36.4	20.7 ± 21.7
Chocolate chip cookies	5.4	63.8	22.6	3.5	0.5	4.87	28	136.4	85.0 ± 20.3	64.2 ± 22.5
Grapes	< 0.5	15	< 0.5	0.7	< 0.5	0.66	65	42.9	77.3 ± 24.6	81.6 ± 15.4
Chocolate wafer bar (KitKat®)	5.9	63.3	25.7	2.1	0.14	5.13	45	230.9	79.9 ± 24.2	68.3 ± 22.2
Potato chips (Hula hoops®)	3.3	63	26.4	2.2	1.8	5.07	24	121.7	76.4 ± 24.5	61.6 ± 21.2
Yogurt (Frube®)	4.9	13.4	2.8	< 0.1	0.13	1.02	40	40.8	66.5 ± 29.5	44.5 ± 25.8

### 2.3 Measures

#### 2.3.1 Energy density preference

The nine snack-food images were displayed on a laptop in a 3 × 3 grid and the position of each food was randomized across participants. To complete the task, participants used the computer mouse to click on each food image in turn. After selecting a food it disappeared from the grid. Participants began by clicking the *most preferred* snack and then repeated this process until no snack foods remained. We calculated an energy density preference (EDP) score using a linear model and regressed the energy density (kcal/g) of the foods onto their respective rank order (1 = least preferred, 9 = most preferred). The EDP score is provided by the slope (β coefficient) that relates energy density to rank order. Positive EDP scores indicate that foods with high energy density were preferred. Negative EDP scores indicate the converse.

We obtained four separate EDP scores from each child-parent dyad; one from each child and three from each parent. Children were asked “Which food would you choose for your lunchbox?” (EDP_child_). Since children tend to select fewer unhealthy foods if they are aware or suspicious that they are being monitored by their parents [27], they were instructed to make their own selections without considering the wishes of their parents. Parents completed the ranking task in response to the following questions: “Which food would you choose for your lunchbox?” (EDP_parent_), “Which food would you *serve* in *your child's* lunchbox?” (EDP_serve_), and “Which food would *your child* choose for *their* lunchbox?” (EDP_estimate_). In the latter scenario, parents were told to imagine that their child had free selection of the foods without parental interference.

#### 2.3.2 Energy density liking

To determine how well-liked the snack foods were, images of the nine foods were presented in succession on the computer screen in standard single-serving portions. Participants were shown a computerized 100-mm visual-analogue scale titled “How much do you LIKE the taste of this food?” with anchor points “I hate it” to “I love it”. Visual-analogue scales have previously been used with children successfully, provided they include age-appropriate modifications. For example, children as young as five were able to rate their emotions (*e.g.*, anxiety, sadness, anger, worry) using 100 mm visual-analogue scales anchored with happy and sad faces [28]. Here, we used child-friendly anchor points (I hate it–I love it) instead of those typically used for adults (Not at all–Extremely). To ensure clarity for the children, the researcher also pointed to each end of the scale and read the corresponding anchor point aloud, then pointed at the scale itself and asked “or somewhere in between?”. Participants rated their liking of each food by using the computer mouse to click on the scale. The order of presentation of the snack food pictures was randomized across participants. Both children and parents rated their own liking of the nine snack foods. To quantify liking for energy-dense foods (EDL), we conducted linear regressions based on the liking ratings (0 = hated, 100 = loved) and the energy density of each food (kcal/g). The resulting β coefficients from the linear regressions were the EDL scores for children (EDL_child)_ and parents (EDL_parent_), respectively. Positive EDL scores indicate that foods with high energy density are liked better. Negative EDL scores indicate the converse.

#### 2.3.3 Familiarity

To assess familiarity with the snack foods, each food image (single-serving portions) was presented on the laptop screen in a randomized order. For each food image, all parents and children were asked “Have you ever eaten this food before?” with possible response options “Yes” or “No.”

### 2.4 Procedure

Parents read an information sheet before providing written consent for themselves and their child to take part. Participants were tested in a private area within the science center. Parents and children completed all computerized measures on separate laptops at opposite ends of a table and were encouraged to not speak to one another during the testing session. We were confident that the children would be able to use our tasks without assistance as this is consistent with previous observations that four-year olds are able to make self-assessments using computerized images [29]. Nonetheless, all instructions, including names of foods where necessary, were read aloud to each child. Children completed measures in the following order; i) EDP_child_, ii) EDL_child_, and iii) familiarity. Parents completed their measures in the following order; i) EDP_serve_, ii) EDP_estimate_, iii) EDP_parent_, iv) EDL_parent_, and v) familiarity. Children's and parents' height was then measured to the nearest millimeter using a portable stadiometer. A single measurement of weight was taken to the nearest 0.1 kg using a Tanita TBF-531 digital scale. Participants were asked to remove shoes and bulky clothing for these anthropometric measurements. Parents also reported their child's date of birth. To account for age and sex differences, child BMI percentiles were computed using a BMI percentile calculator for children and teens, provided by the Center for Disease Control and Prevention [30]. Parent BMI was calculated as kg/m^2^. Finally, parents were given a debriefing sheet which explained the broad aims of the research and participants were thanked for their assistance. Each session lasted approximately fifteen minutes.

### 2.5 Data analysis

Several participants (children *n* = 13, parents *n* = 3) were unfamiliar with three or more of the snack foods and were excluded on this basis. Some participants did not provide data for all tasks (children *n* = 2, parents *n* = 1) and were also removed. Finally, we excluded data from one child who had an EDL_child_ score that was more than five standard deviations away from the mean, leaving 110 child-parent dyads in the final data set.

To evaluate evidence for discrimination between foods based on energy density, we conducted separate 1-sample *t*-tests to determine whether the EDP scores (child, parent, serve, estimate) and EDL scores (child, parent) deviate significantly from zero. In the first instance we calculated bivariate correlations between child BMI percentile and the following variables: EDL_child_, EDP_child_, EDL_parent_, EDP_parent_, EDP_serve,_ EDP_estimate_ and parental BMI. To consider their combined role as predictors of child BMI percentile we then assessed three separate models using simultaneous linear regression. Respectively, the first and second of these assessed the measures from children (EDL_child_, EDP_child)_ and from parents (EDL_parent_, EDP_parent_, EDP_serve,_ and EDP_estimate_). In the final model we entered all variables together with parental BMI. Differences were considered significant at *p* < 0.05 and all results are reported as means ± *SD*. All analyses were conducted using SPSS version 23.0.0.2 (SPSS Inc., Chicago, IL, USA.).

## 3 Results

Our final sample (*N* = 110) was well-balanced for gender across the child-parent dyads and included female-female (*n* = 34), female-male (*n* = 23), male-male (*n* = 22), and male-female (*n* = 31) pairs respectively. Further participant characteristics can be found in Table 2. In addition, all snack foods were generally familiar and were reasonably liked, both by children and parents (see Table 1).

**Table 2:** Participant characteristics (*N* = 110). Values for age, BMI and BMI percentile are means and standard deviations. Values for gender are frequencies.

	Children	Parents
Age (years)	8.4 ± 2.7, Range: 3–14	–
BMI (percentile)	59.2 ± 25.6, Range: 4–98	–
BMI (kg/m^2^)	–	26.3 ± 4.5, Range: 17.7–43.2
Gender (% female)	51.8	59.1

EDP_child_ and EDL_child_ scores tended to be positive and deviated significantly from zero, suggesting that children preferred and liked higher energy-dense foods. Significant and positive EDP_estimate_ scores indicated that parents correctly estimated this outcome. All other scores from parents (EDP_parent_, EDL_parent_, and EDP_serve_) tended to be negative and also deviated significantly from zero, suggesting that lower energy-dense foods are preferred, liked, and served by parents (see Table 3).

**Table 3:** Mean EDP and EDL scores (*N* = 110, *df* = 109) together with associated summary statistics evaluating deviation from zero.

	Mean	*SD*	*t*	95% CI	*p*
EDP_child_	0.12	0.33	3.91	(0.06, 0.18)	< 0.001
EDP_parent_	− 0.12	0.28	− 4.63	(− 0.18, − 0.07)	< 0.001
EDP_serve_	− 0.36	0.25	− 15.06	(− 0.41, − 0.32)	< 0.001
EDP_estimate_	0.21	0.3	7.18	(0.15, 0.26)	< 0.001
EDL_child_	0.01	0.04	3.28	(0.00, 0.02)	0.001
EDL_parent_	− 0.01	0.03	− 2.75	(− 0.01,− 0.00)	0.007

Bivariate correlations (see Table 4) showed no significant relationship between child BMI percentile and any of the EDP or EDL scores. As expected, parental BMI and child BMI percentile were highly correlated (*r* = 0.37, *p* < 0.001).

**Table 4:** Pearson's correlations (*r*) to assess relationships between variables. Associated *p* values are presented in brackets (*N* = 110).

	Parent BMI	EDP_child_	EDL_child_	EDP_parent_	EDL_parent_	EDP_serve_	EDP_estimate_
Child BMI percentile	0.368 (< 0.001)	− 0.088 (0.361)	− 0.083 (0.391)	− 0.088 (0.363)	0.030 (0.756)	0.064 (0.510)	0.002 (0.981)
Parent BMI	–	− 0.054 (0.573)	0.069 (0.472)	− 0.150 (0.118)	− 0.199 (0.037)	− 0.087 (0.366)	− 0.102 (0.288)
EDP_child_	–	–	0.473 (< 0.001)	0.132 (0.169)	0.054 (0.573)	0.120 (0.211)	0.205 (0.031)
EDL_child_	–	–	–	0.058 (0.548)	− 0.032 (0.742)	0.117 (0.224)	0.104 (0.277)
EDP_parent_	–	–	–	–	0.551 (< 0.001)	0.125 (0.193)	0.264 (0.005)
EDL_parent_	–	–	–	–	–	0.063 (0.515)	0.093 (0.333)
EDP_serve_	–	–	–	–	–	–	0.257 (0.007)

The variables included in the first and second linear regression models (Table 5a and b) failed to explain a significant proportion of variance in child BMI percentile. To address the possibility that performance on the tasks varied as a function of age, *post hoc* we added interaction terms for children's EDP and EDL scores adjusted for child age to the first regression model. We calculated the interaction terms by multiplying the standardized values (z-scores) of both EDP_child_ and EDL_child_ by child age (in years). All significant and non-significant effects remained unchanged (results not shown). The average EDP and EDL scores for children are reported in Table 6, partitioned by child age.

**Table 5:** Linear regressions predicting child BMI percentile.

	Mean (*SD*)	*B*	*β*	95% CI	Model fit
a. Model 1 - EDP_child_, EDL_child_					
EDP_child_	0.12 (0.33)	0.55	0.01	(− 16.48, 17.59)	R^2^ = 0.000, adjusted R^2^ = − 0.019, *R* = 0.006, intercept = 59.107, *p* = 0.998
EDL_child_	0.01 (0.04)	− 1.78	0.00	(− 156.73, 153.18)	

b. Model 2 - EDP_parent_, EDL_parent_, EDP_serve_, EDP_estimate_					
EDP_parent_	− 0.12 (0.28)	− 11.57	− 0.13	(− 32.88, 9.74)	R^2^ = 0.071, adjusted R^2^ = 0.035, *R* = 0.266, intercept = 48.272, *p* = 0.100
EDL_parent_	− 0.01 (0.03)	37.03	0.41	(− 165.82, 239.88)	
EDP_serve_	− 0.36 (0.25)	− 24.48	− 0.24	(− 43.97, − 4.99)	
EDP_estimate_	0.21 (0.30)	3.91	0.05	(− 12.97, 20.79)	

c. Model 3 - EDP_child_, EDL_child_, EDP_parent_, EDL_parent_, EDP_serve_, EDP_estimate_, Parent BMI					
EDP_child_	0.12 (0.33)	4.88	0.06	(− 11.03, 20.78)	R^2^ = 0.206, adjusted R^2^ = 0.151, *R* = 0.454, intercept = − 7.907, *p* = 0.001
EDL_child_	0.01 (0.04)	− 17.73	− 0.03	(− 160.76, 125.30)	
EDP_parent_	− 0.12 (0.28)	− 10.82	− 0.12	(− 30.89, 9.25)	
EDL_parent_	− 0.01 (0.03)	94.56	0.10	(− 98.19, 287.30)	
EDP_serve_	− 0.36 (0.25)	− 22.54	− 0.22	(− 40.95, − 4.13)	
EDP_estimate_	0.21 (0.30)	5.20	0.06	(− 10.87, 21.27)	
Parent BMI	26.35 (4.48)	2.15	0.38	(1.12, 3.19)

**Table 6:** Average EDP and EDL scores partitioned by child age (years). Values are displayed as means and standard deviations (*N* = 110).

Child age (years)	*n*	EDP_child_	EDL_child_
3	1	0.23 (−)	0.024 (−)
4	4	0.15 (0.06)	0.013 (0.019)
5	13	0.21 (0.32)	0.014 (0.032)
6	15	0.13 (0.33)	0.008 (0.023)
7	14	0.22 (0.38)	0.014 (0.029)
8	7	0.01 (0.45)	0.003 (0.034)
9	12	0.10 (0.33)	0.021 (0.060)
10	17	0.05 (0.31)	0.020 (0.026)
11	10	0.13 (0.30)	0.006 (0.050)
12	11	0.04 (0.35)	0.002 (0.035)
13	4	0.12 (0.36)	− 0.011 (0.055)
14	2	0.30 (0.07)	0.011 (0.011)

The final linear regression (see Table 5c) produced a significant model and accounted for 20.6% of the variance in child BMI percentile. However, this was largely explained by variation in parent's own BMI. In this final model, EDP_serve_ was a significant predictor (*p* = 0.017), but in a counterintuitive direction – parents with lean children had a greater tendency to prioritize higher energy-dense foods when selecting foods to serve to their child. In combination, these observations provide no clear evidence that child BMI percentile is associated with child or parent preference for energy-dense foods.

## 4 Discussion

In this study, we considered two specific propositions that might explain the relationship between dietary energy density and child BMI percentile. First, we explored whether child BMI percentile is associated with individual differences in children's relative preference for energy dense foods. Higher energy-dense foods tended to be selected and better liked by children, however, individual variation in these preferences was not associated with their BMI percentile. Second, we examined whether the same individual differences in parents were associated with their child's BMI percentile. Overall, there was little evidence of this relationship and, as in previous studies [31], [32], parent BMI was the main predictor of child BMI percentile. Based on evidence suggesting a positive association between dietary energy density and adiposity in children [8], we anticipated that parents with a tendency to serve energy-rich foods might have children with higher BMI. Our data indicate the converse - a negative association. We see two potential reasons why this might be the case. First, the relationship reflects inaccurate reporting resulting from a ‘desirability bias’ [33]. Second, the relationship accurately reflects parental choices, which are governed by a concern to reduce their child's BMI by serving lower energy-dense foods. In other words, the EDP_serve_ task reflects feeding behavior that is responsive to children's current weight rather than capturing behaviors that promoted initial weight gain. To distinguish between these accounts, measures are needed of the foods that are actually served to children and on a longitudinal basis.

Although our data provide little evidence that variation in relative preference for energy dense foods is associated with child BMI percentile, previous evidence has shown that taste preferences may differ by weight status in both children and adults. For example, overweight and obese children appear to show a greater preference for the taste of fat and sweetness [34]. Similarly, children with overweight parents have been shown to prefer high-fat foods [35]. Since fats and sugars are key contributors to the energy density of foods, increased preference for their taste may promote their consumption. However, we are unaware of any previous assessment of our specific hypothesis, that relative preference for energy density is associated with BMI.

Our data should not be taken to dispute the epidemiological evidence that dietary energy density is associated with childhood BMI. Rather, they indicate that factors other than relative preference for energy density are likely to explain this relationship. Before children gain full dietary autonomy, parents have control over much of their child's diet. Therefore, availability is likely to be a key determinant of the consumption of energy rich foods and individual parents are likely to vary in the extent to which they make these foods accessible to their children. For example, energy-dense foods are less expensive, which encourages their consumption in low-income households [36] where the cost of purchasing healthier, low energy-dense options is a barrier [37], [38]. Conversely, children may have reduced access to high-energy-dense foods based on parents' tendency to restrict their consumption. Parental influence on child eating behavior, including the restriction of specific foods, has been studied extensively [39]. Restriction might be associated with parents' own difficulty in controlling food intake, perceptions about their child's ability to self-regulate, and concerns about their child's risk of developing problematic eating behavior [40]. The decision to limit energy-dense food consumption may also be based on parents' perceptions about their child's weight [41], [42].

Our study considers the effect of energy density on food choice in isolation. However, we note that choice is governed by an interaction between energy density and portion size [4], [5]. Therefore, future studies might incorporate an assessment of the portions of snack foods that are selected. Further, there is an interplay between palatability and expected satiety when selecting foods that vary in portion size [43]. Understanding the influence of expected satiety in the context of our lunchbox task could be valuable. A potential limitation to the current task is that we did not capture participants' motivation behind their selections. Future research could extend the range of parental measures to examine whether choices are motivated by beliefs about the healthiness and appropriateness of snack foods that vary in energy density.

Our task assessed only the behavioral tendency to *select* energy-dense foods. As such, evidence that energy-dense foods were preferred should not be taken to imply that participants applied a conceptual understanding of energy density and/or nutrient composition. It is well established that humans rely on range of cues (*e.g.*, learned flavor-nutrient associations) to differentiate foods based on their energy density and macronutrient composition [44]. Indeed, rodents and other omnivores use the same cues and do so in the absence of explicit nutritional information (*e.g.*, labelling). Regardless of participants' explicit understanding of energy density, concerns about whether the task is sensitive enough to detect a preference for energy density are allayed by the observed and predicted positive relationships between choice and energy density, both in EDP_child_ and EDP_estimate_ measures.

We note that there are several other limitations to the current study which could be addressed by further research. In particular, the task could be applied to a broader range of scenarios beyond those currently included. We selected a lunchbox scenario because it was likely to be familiar to most children within the selected age range. However, we acknowledge that the foods typically consumed by children within this age range could vary widely and some children may have little experience with packed lunches. Also, we note that there may be conceptual limitations around the lunchbox scenario (*e.g.* school rules surrounding permissible lunchbox foods) that may have impacted responses on this task. Further, the foods in the current study were selected because they are convenient (*i.e.* portable, inexpensive) lunchbox foods. However, the task could be extended by using a broader range of snack foods and main meals that are appropriate for both children and adults, and also by indexing their prior exposure to these foods. Future studies that employ our task with child-parent dyads should ensure that the parent participant is also the primary meal provider. Here, although we asked parents their primary caregiver status, we do not know whether they were also the primary provider of meals to their child. This could impact parents' performance in estimating their children's preferences and ability to report what they would typically serve to their child. Despite these limitations, the key strength of our novel computer-based task is its simplicity and even young children found it easy to complete with minimal assistance. This enabled us to make a direct comparison between EDP and EDL scores across children and their parents. We hope that by demonstrating the successful implementation of our paradigm others might consider its application in related studies.

## Conflict of interest

The authors report no conflict of interest related to this study.
